# Accuracy of Robotic and Frame-Based Stereotactic Neurosurgery in a Phantom Model

**DOI:** 10.3389/fnbot.2022.762317

**Published:** 2022-03-25

**Authors:** Andrea Spyrantis, Tirza Woebbecke, Daniel Rueß, Anne Constantinescu, Andreas Gierich, Klaus Luyken, Veerle Visser-Vandewalle, Eva Herrmann, Florian Gessler, Marcus Czabanka, Harald Treuer, Maximilian Ruge, Thomas M. Freiman

**Affiliations:** ^1^Department of Neurosurgery, Center of Neurology and Neurosurgery (ZNN), University Hospital Frankfurt, Goethe-University, Frankfurt am Main, Germany; ^2^Department of Stereotactic and Functional Neurosurgery, Faculty of Medicine, University Hospital Cologne, University of Cologne, Cologne, Germany; ^3^Institute of Biostatistics and Mathematical Modeling, Goethe University, Frankfurt am Main, Germany; ^4^Department of Neurosurgery, University Medical Center Rostock, Rostock, Germany

**Keywords:** stereotactic neurosurgery, robot-guided stereotaxy, phantom study, mechanical accuracy, stereotactic frame

## Abstract

**Background:**

The development of robotic systems has provided an alternative to frame-based stereotactic procedures. The aim of this experimental phantom study was to compare the mechanical accuracy of the Robotic Surgery Assistant (ROSA) and the Leksell stereotactic frame by reducing clinical and procedural factors to a minimum.

**Methods:**

To precisely compare mechanical accuracy, a stereotactic system was chosen as reference for both methods. A thin layer CT scan with an acrylic phantom fixed to the frame and a localizer enabling the software to recognize the coordinate system was performed. For each of the five phantom targets, two different trajectories were planned, resulting in 10 trajectories. A series of five repetitions was performed, each time based on a new CT scan. Hence, 50 trajectories were analyzed for each method. X-rays of the final cannula position were fused with the planning data. The coordinates of the target point and the endpoint of the robot- or frame-guided probe were visually determined using the robotic software. The target point error (TPE) was calculated applying the Euclidian distance. The depth deviation along the trajectory and the lateral deviation were separately calculated.

**Results:**

Robotics was significantly more accurate, with an arithmetic TPE mean of 0.53 mm (95% CI 0.41–0.55 mm) compared to 0.72 mm (95% CI 0.63–0.8 mm) in stereotaxy (*p* < 0.05). In robotics, the mean depth deviation along the trajectory was −0.22 mm (95% CI −0.25 to −0.14 mm). The mean lateral deviation was 0.43 mm (95% CI 0.32–0.49 mm). In frame-based stereotaxy, the mean depth deviation amounted to −0.20 mm (95% CI −0.26 to −0.14 mm), the mean lateral deviation to 0.65 mm (95% CI 0.55–0.74 mm).

**Conclusion:**

Both the robotic and frame-based approach proved accurate. The robotic procedure showed significantly higher accuracy. For both methods, procedural factors occurring during surgery might have a more relevant impact on overall accuracy.

## Introduction

The development of robotic techniques is challenging the gold-standard of frame-based stereotactic procedures in neurosurgery. Both methods are established for deep brain stimulation, stereoencephalography and biopsies of intracranial lesions (Neudorfer et al., 2018; Faraji et al., 2020). Numerous studies on robotics in neurosurgery are available, confirming a high level of accuracy and safety (Lefranc et al., 2015; de Benedictis et al., 2017; Fomenko and Serletis, 2018; Marcus et al., 2018; Spyrantis et al., 2018, 2019; Philipp et al., 2021). For biopsies, complication rates and the diagnostic yield are comparable (Livermore et al., 2014; Lefranc et al., 2015; Yasin et al., 2019).

Accuracy mainly depends on the referencing technique (Lefranc et al., 2014; Spyrantis et al., 2019), but also on the patient's anatomy, the quality of planning data as well as other factors (Maciunas et al., 1992; Cardinale, 2015; Liu et al., 2020; Lu et al., 2021). Several phantom studies on robotic stereotactic devices confirmed the clinical data on differences in accuracy depending on the referencing technique (Li et al., 2002; Lefranc et al., 2014; von Langsdorff et al., 2015; Cardinale et al., 2017).

In robotic surgery using the ROSA system, referencing may be accomplished via a laser scan of the face, referenced to either the planning MRI data or an additional 3D head computed tomography (CT) scan fused with the planning MRI data. Highest accuracy is accomplished with a Leksell frame reference (Spyrantis et al., 2019). In a phantom study, Lefranc et al. confirm a higher target accuracy of the ROSA system with CT-based frameless referencing or frame-based referencing (0.3 mm) compared to frameless MRI-based referencing (1.59 mm). These findings were affirmed by a retrospective series of frame-based and frameless stereotactic surgery procedures (Lefranc et al., 2014). Liu et al. demonstrated accurate deep brain stimulation (DBS) lead implantation with the ROSA system using frameless, fiducial-based referencing with a lateral deviation in the sub-millimeter range (Liu et al., 2020). Another study comparing frameless referencing methods in different clinical applications was published by Brandmeir et al. (2018). Accuracy of facial laser scan referencing and fiducial-based referencing did not show a statistically significant difference, with the mean TPE ranging from 3.9 to 4.5 mm.

In frame-based stereotactic surgery, referencing is accomplished using a 3D CT scan with the Leksell frame fixed to the patient's head and later fused to the planning MRI data.

This study aims to evaluate the mechanical accuracy of the robotic technique as an alternative to manual frame-based stereotaxy. To achieve this assessment without the effects of the referencing method on accuracy, the highly accurate Leksell frame referencing was chosen for both methods. The experimental approach using a phantom excludes various unpredictable confounders of the clinical setting.

## Materials and Methods

The Leksell Stereotactic System® (Elekta Stockholm, Sweden) with the Leksell Coordinate Frame G® was chosen for referencing in both methods. All experiments were performed using the same frame to exclude mistakes due to different age-dependent deterioration of the material.

**Figure 1:**
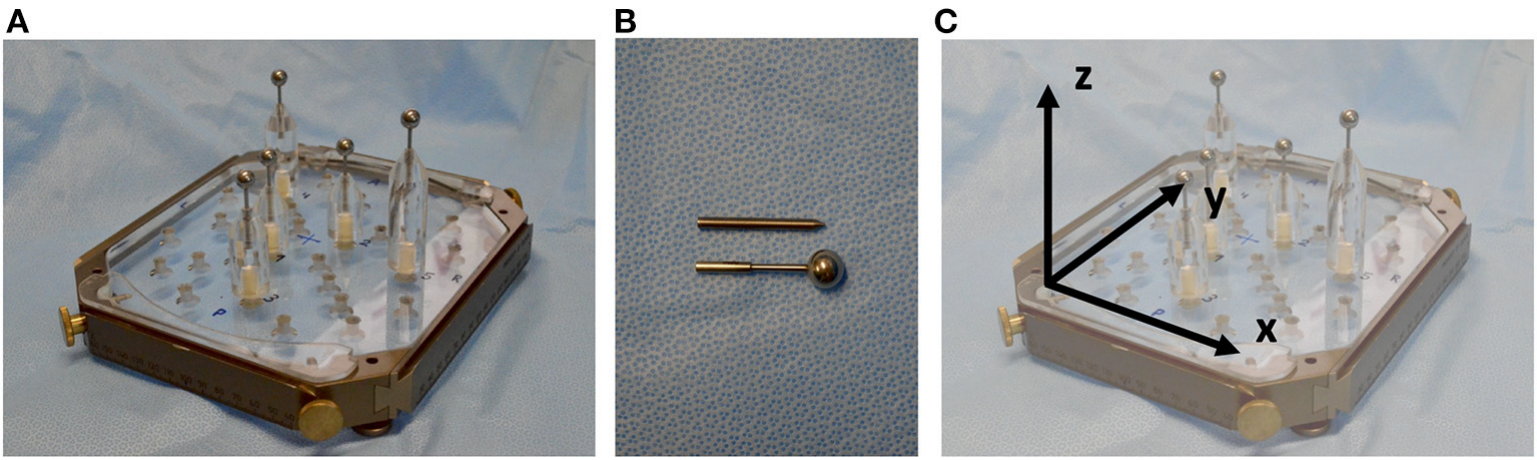
**(A)** PMMA phantom with support brackets and spherical target rods fitted into the Leksell frame. **(B)** Spherical rods were used for target point definition in planning and needle tip rods were used for target point determination and error assessment after positioning the probe along the trajectory. **(C)** The reference system is defined by the transversal (x-), the longitudinal (y-) and the sagittal (z-) axis.

An acrylic phantom (Polymethylmetacrylat, PMMA), designed to fit into the Leksell frame, was used (Figure 1A). For this phantom, plastic support brackets of variable heights holding metal target rods can be attached in different positions. For the identification of the target point in the CT scan, spherical rods were used, whereas for the assessment of the target point error, rods with a needle tip were used (Figure 1B). The phantom's reference system is defined by the transversal (x-), the longitudinal (y-) and the sagittal (z-) axis (Figure 1C).

**Figure 2:**
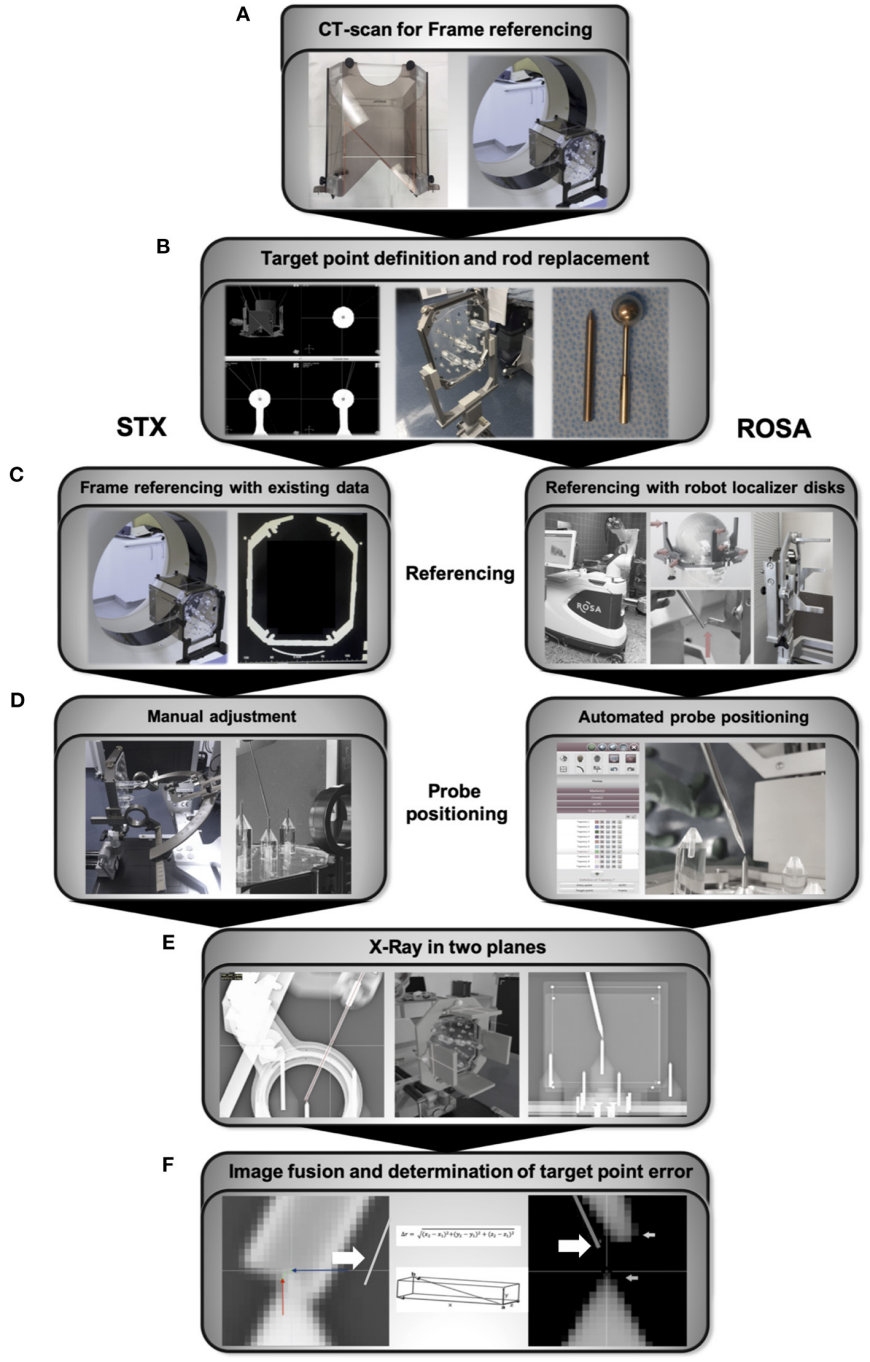
Workflow **(A)** CT scan with the Open CT Indicator attached to the frame for referencing and generation of planning data. **(B)** Manual identification of the sphere center and trajectory planning, followed by replacing the spherical rods for the tip rods. **(C)** Referencing of the Leksell frame was already accomplished with the initial referencing and planning CT scan. Referencing of the robotic system via localizer disks attached to the Leksell frame. The robotic arm is therefore manually guided to nine indentations on three localizer disks fixed to the frame. **(D)** The x-, y-, z- coordinates, the arc and ring angle of the planned trajectories are calculated by the IPS planning software for the frame-based stereotactic experiment and manually transferred to the frame and the attached Leksell Multi-Purpose Stereotactic Arc. In the robotic experiment, the robotic arm automatically drives to the planned trajectory. **(E)** The final position of the probes positoned along the trajectories was controlled with X-ray in two planes, anterior-posterior and lateral, using a permanently installed X-ray unit. **(F)** For both experiments, X-rays were uploaded to the ROSA-software and fused with the planning data depicting the planned trajectories (bold arrow). Coordinates of the 1.6 mm diameter probe's center at the endpoint and the tip of the spike-shaped probe, respectively, were visually determined and used to calculate the ED, the depth deviation, the lateral deviation and the deviations in all three axes. STX, frame-based experiment.

A CT Scan with the phantom fixed to the Leksell frame and a localizer (Open CT Indicator) enabling the software to calculate and reference the coordinate system was performed. The Open CT Indicator (Figure 2A) is equipped with “N”-shaped radiopaque strips on both sides as well as on the front disk, enabling the software to determine its exact position. 0.67 mm slice CT scans were performed using the Philipps iCT 256 (Philipps, Best, Netherlands, pixel size 0.63 mm, matrix size 512 × 512). Data were transferred to the planning software iPS (inomed Planning System, inomed Medizintechnik GmbH, Emmendingen, Germany) (Figure 2A).

For trajectory planning, the center of the spherical localizing objects was manually determined and the coordinates registered (Figure 2B). Only the target point, but no entry point was planned as the phantom is not equipped with a shell simulating the head's surface. While positioning the probe, needle tip rods were used to depict the target point (Figure 2B). Those are 0.5 mm longer than the center of the spherical probes. For planning, 0.5 mm were therefore added to the z-coordinate of the targets. For each of the 5 phantom targets, 2 different trajectories were planned, resulting in 10 trajectories. To simulate the aberrations inherent to fixing the Leksell frame to a patient's head, a series of 5 repetitions were performed, each time based on a new CT scan after removing and repositioning the phantom in the Leksell frame and reattaching the frame to its bracket. In total, 50 trajectories were analyzed for each method.

### Robotic Experiment

The stereotactic frame holding the phantom was attached to the Robotic Surgery Assistant (ROSA Brain 2.5, Zimmer Biomet Robotics, formerly MedTech, Montpellier, France), spherical localizing objects were replaced by needle tips. Referencing was accomplished by manually guiding the robotic arm to special localizer disks attached to the Leksell frame (Figure 2C). The deviation of the registered coordinates and the actual coordinate system is defined as registration error, which is assessed by the ROSA software and either accepted or declined (when exceeding a certain deviation).

Via the robotic control unit, the ROSA arm was moved in line with the planned trajectories to the predefined targets on the stereotactic phantom (Figure 2D). After replacing the robot localizer disks with X-ray localizer disks (Zimmer Biomet, Montpellier, France), the final position of the spike-shaped probe attached to the robotic arm was controlled with X-ray in two planes, anterior-posterior and lateral, using a permanently installed X-ray unit (Brandis Medizintechnik GmbH, Weinheim, Germany, pixel size 0.175 mm, matrix size 1994 × 2430) (Figure 2E). Before moving to the next trajectory, the robotic arm was guided back to the starting position.

### Frame-Based Experiment

For the frame-based experiment, x-, y-, z- coordinates, arc angle and ring angle are calculated by the planning software iPS, defining the target point of each trajectory. These coordinates and angles are manually transferred to the stereotactic system with the Leksell Multi-Purpose Stereotactic Arc attached to the Leksell frame, which was firmly fixed and stabilized. Adjustments were verified by a second investigator. Experiments were performed using the Insertion Cannula kit (190 mm cannula, 1.6 mm in diameter) and same stereotactic phantom, positioning was controlled with X-ray using the localizer disks described above (Figure 2E).

### Analysis

For both experiments, the X-rays depicting the final probe position and its distance to the target were fused with the planning data (Figure 2F). The coordinate system for each acquired fluoroscopic image set was defined by the X-ray localizer disks fixed to the Leksell-frame. Deviations were determined by the ROSA software (ROSA 2.5.8 Brain Application, Zimmer Biomet Robotics, formerly MedTech, Montpellier, France) and accepted for values <0.5 mm.

**Figure 3:**
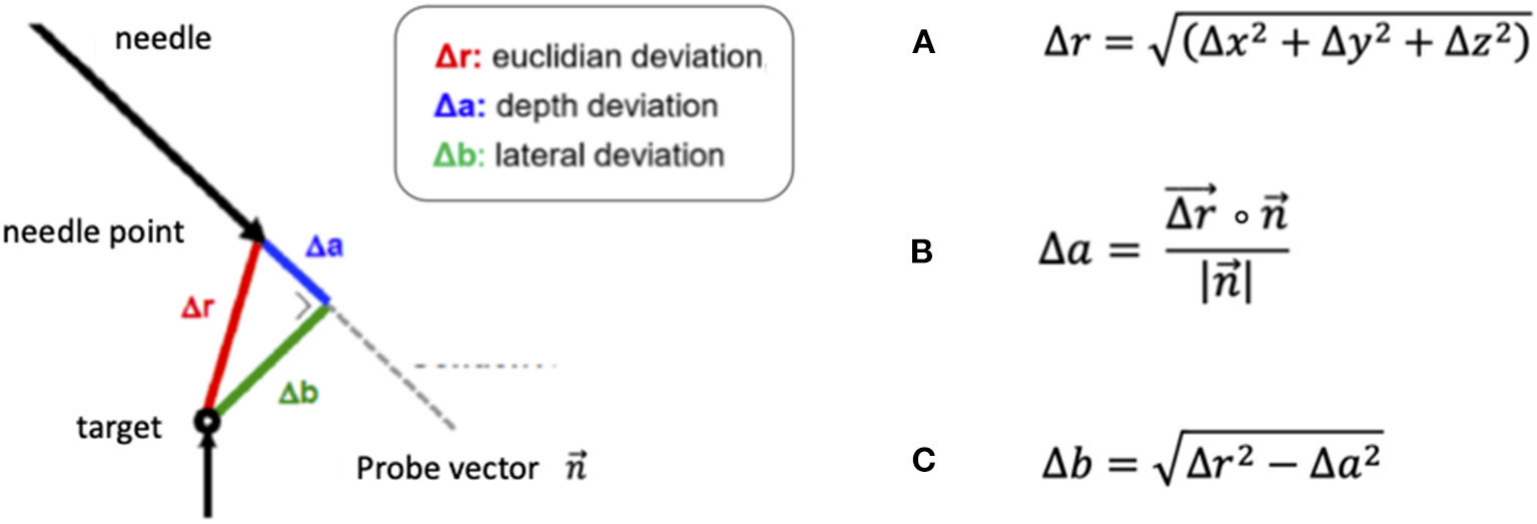
Formulas for the calculation of **(A)** the Euclidian distance (ED) Δ*r*, **(B)** the depth deviation Δ*a*, defined as the scalar product of the ED and the probe vector divided by the amount of the probe vector and **(C)** the lateral deviation Δ*b*, based on the previously calculated depth deviation.

The coordinates of the target point, depicted in the X-ray imaging as the tip of the needle rod positioned in the phantom, and the endpoint of the robot- or frame-guided probe (Figure 2F) were visually determined using the ROSA software. In the robotic experiment, the endpoint was defined as the tip of the spike-shaped probe whereas in the frame-based experiment, the endpoint was defined as the centre of the 1.6 mm diameter probe. With these coordinates, the Euclidian distance (ED, Figure 3A) was calculated, defining the distance between the fluoroscopically determined coordinates and therefore the target point error (TPE).

In addition, we separately calculated the depth deviation (Figure 3B) along the trajectory, the lateral deviation (Figure 3C) and individually analyzed the deviation in all three axes: the transversal (x-) axis, the longitudinal (y-) axis and the sagittal (z-) axis.

Statistical analysis was performed using GraphPad Prism 8. Normal distribution was tested applying the Kolmogoroff-Smirnoff-Lilliefors test (KLS-Test). Data was analyzed using the Kruskal-Wallis test, *p* < 0.05 was considered significant. Further analysis comparing the accuracy in the different test series was performed using the Dunn's test. For figure editing, Microsoft PowerPoint for Mac Version 16.16.27 was used.

## Results

### Robotic Experiment

Robotic accuracy was determined, with an arithmetic mean of the TPE of 0.53 mm (SD ± 0.27 mm, 95% CI 0.41–0.55 mm, Table 1). The depth deviation along the trajectory was calculated using the scalar product of the Euclidian distance and the vector of the actual trajectory, the probe vector $\overset{\to }{n}$ (Figure 3B). Negative values represent a position before reaching the target point, positive values a position beyond the target point. The mean depth deviation along the trajectory was −0.22 mm (SD ± 0.24 mm, 95% CI −0.25 to −0.14 mm, Table 2). The mean lateral deviation amounted to 0.43 mm (SD ± 0.26 mm, 95% CI 0.32–0.49 mm, Table 3). The mean deviation in all three axes was determined with −0.01 mm (SD ± 0.34 mm, 95% CI −0.11–0.09 mm) in the transversal axis and more prominent differences of 0.22 mm (SD ± 0.24 mm, 95% CI 0.15–0.29 mm) in the longitudinal axis and 0.23 mm (SD ± 0.29 mm, 95% CI 0.15–0.31 mm) in the sagittal axis (Table 4).

**Table 1:** Target point error in Leksell-frame and ROSA robot.

**Target point error**	**Leksell®-Frame**	**ROSA®-Robot**
Arithmetic mean [mm]	0.72	0.53^*^
Standard deviation [mm]	0.31	0.27
95%-CI [mm]	0.63–0.81	0.41–0.55

* *Statistically significant*.

**Table 2:** Depth deviation in Leksell-frame and ROSA robot.

**Depth deviation**	**Leksell®-Frame**	**ROSA®-Robot**
arithmetic mean [mm]	−0.2	−0.22
standard deviation [mm]	0.21	0.24
95%-CI [mm]	−0.26 to−0.14	−0.25 to−0.14

**Table 3:** Lateral deviation in Leksell-frame and ROSA robot.

**Lateral deviation**	**Leksell®-Frame**	**ROSA®-Robot**
Arithmetic mean [mm]	0.65	0.43^*^
Standard deviation [mm]	0.33	0.26
95%-CI [mm]	0.55–0.74	0.32–0.49

* *Statistically significant*.

**Table 4:** Transversal-, longitudinal and sagittal deviation in Leksell-frame and ROSA robot.

**Directional error arithmetic mean [mm]**	**Leksell®-Frame**	**ROSA®-Robot**
Transversal deviation	−0.21	−0.01
Longitudinal deviation	0.18	0.22
Sagittal deviation	−0.22	0.23^*^

* *Statistically significant*.

### Frame-Based Experiment

The TPE in the frame-based setting was determined with a mean of 0.72 mm (SD ± 0.31 mm, 95% CI 0.63–0.8 mm). The mean depth deviation amounted to −0.20 mm (SD ± 0.21 mm, 95% CI−0.26 to −0.14 mm), the mean lateral deviation to 0.65 mm (SD ± 0.33 mm, 95% CI 0.55–0.74 mm). Depth deviation was negative in most cases.

Further analysis of the directional error showed a mean deviation in the transversal axis of −0.21 mm (SD ± 0.37 mm, 95% CI −0.31 to −0.11 mm), a mean longitudinal deviation of 0.18 mm (SD ± 0.20 mm, 95% CI 0.12–0.24 mm) and a mean deviation of −0.22 mm (SD ± 0.57 mm, 95% CI −0.38 to −0.06 mm) in the sagittal axis.

### Comparing Accuracy of the ROSA Robot and Frame-Based Stereotaxy

With an arithmetic mean TPE of 0.53 mm, the robotic procedure was more accurate than the manually adjusted, frame-based stereotactic procedure with a mean TPE of 0.72 mm, the difference was statistically significant (*p* = 0.0012, Table 1). There was no statistically significant difference in depth deviation (−0.22 mm robotics vs. −0.2 mm in stereotaxy, Table 2), but a statistically significant difference in lateral deviation, which was higher in stereotaxy (0.43 mm in robotics vs. 0.65 mm in stereotaxy, Table 3).

Deviations in the transversal, longitudinal and sagittal axes were compared in both methods. Deviation did not differ significantly in the transversal (*p* = 0.0598) or longitudinal axis (*p* = 0.59). There was a statistically significant difference in deviation in the sagittal axis (*p* < 0.0001). With the mean sagittal deviation in stereotaxy being −0.22 mm and 0.23 mm in the robotic experiment, the difference is caused by basically equal deviations in opposite directions (Table 4).

## Discussion

An experimental evaluation of robotic and frame-based mechanical accuracy was accomplished in this phantom study. For both the robotic stereotactic experiment and the Leksell frame-based stereotactic experiment, we used the same phantom, the same referencing method, the same CT scanner and the same X-ray device. Analysis of accuracy showed a very high mechanical accuracy in the sub-millimeter range for both methods with a mean TPE of only 0.53 mm in the robotic approach and a mean TPE of 0.72 mm in classic frame-based stereotaxy.

The significant difference in the actual target point error cannot be explained with an error in one specific axis. The overall lateral deviation, describing a lateral error from the planned trajectory in any direction, was significantly higher in the frame-based procedure (0.65 mm) than in the robotic procedure (0.43 mm), whereas depth deviation did not show a significant difference. The mean depth deviation in our experiments was negative, meaning the probe positioned along the trajectories did in most cases not reach the actual target point. Interestingly, we observed this in both the robotic experiment, with the automated robotic movement guiding the probe, as well as the frame-based experiment, which requires manual adjustment of the probe along the trajectory. It is therefore difficult to determine whether we are looking at a systemic error in the experimental setup or not, an explanation is yet to be found. Previous studies have addressed inaccuracies in frame-based stereotactic procedures due to asymmetrical mounting or distortions of the stereotactic device (Treuer et al., 2004; Alptekin et al., 2019; Renier and Massager, 2019). In the present phantom study, only the manual attachment of the stereotactic arc and the trajectory adjustment differed from the robotic procedure, not the positioning of the frame. The results indicate a loss of precision due to manual adjustments but cannot point out a cause for specific directional errors.

There are several phantom studies analyzing the accuracy of frame-based stereotactic devices. Maciunas et al. performed a phantom study comparing the accuracy of four different stereotactic frames (Galloway et al., 1991). Imaging for target point definition was accomplished with a 1 mm layered CT scan. The mean accuracy determined for the Leksell frame in this study was 1.7 mm (*n* = 900, SD ± 1 mm, Min: 0.2 mm, Max: 4.9 mm), which is less accurate than both the robotic accuracy and frame-based accuracy determined in our phantom study. Possible explanations are a higher resolution of the 0.67 mm slice CT scan we used in the present study as well as precise radiographic determination of deviations in a permanently installed X-ray unit.

Also, robotic stereotactic devices were analyzed in phantom studies before. Cardinale et al. performed a phantom study analyzing the Neuromate robotic system (Renishaw Mayfield SA, Nyon, Switzerland) (Cardinale et al., 2017). With a 0.833 mm layer thickness imaging (O-arm), the TPE amounted to 0.67 mm using a neurolocation device and to 0.76 mm using the Talairach frame for referencing. Li et al. compared the accuracy of the Neuromate robot using different referencing methods. They showed only slightly higher target point errors than Cardinale et al. when determining the accuracy of the Neuromate robot in a phantom study using a 2 mm slice CT scan for frame referencing. The average error for frame-based registration of the Neuromate robot amounted to 0.86 mm (Li et al., 2002).

von Langsdorff et al. (2015) determined the accuracy of the Neuromate robot on a phantom as well as in patients with deep brain stimulation, applying frame-based referencing. In the phantom experiment, the TPE amounted to 0.44 mm, which is comparable to the accuracy of 0.53 mm we determined for frame-based referencing of the ROSA robot in the present phantom study.

With the exception of the study performed by Maciunas et al., these phantom studies report errors of <1 mm, comparable to the accuracy we found in the robotic and Leksell frame-based experimental procedures.

Another phantom study determining the accuracy of the ROSA robot using different referencing methods was performed by Lefranc et al. (2014). Frame-based (*n* = 20) as well as frameless CT based referencing (*n* = 20) accomplished a target accuracy of 0.3 mm compared to 1.59 mm in frameless MRI guided referencing (*n* = 20). Conform with the results in this experimental setting, our previous study on procedural accuracy with different referencing methods in patients with robotic stereo electroencephalography (sEEG) showed a superiority of CT based referencing compared to MRI based referencing. Both frame-based referencing (TPE 2.28 mm) and laser scan-based referencing of the face with a thin layer CT scan fused to the planning MRI (TPE 2.41 mm) were significantly more accurate than laser scan referencing based on the planning MRI alone (TPE 3.51 mm) (Spyrantis et al., 2019). A more accurate frameless registration was described by Liu et al. (2020) who published a study analyzing *in vivo* accuracy of frameless bone fiducial registration in ROSA robot-assisted DBS. Depending on the method chosen for radiographic detection of the lead position, the accuracy ranged from 0.57 to 1.1 mm.

Relevant procedural factors influencing accuracy are found within the clinical application of the devices. The mechanical distortion during frame fixation, for example, results in inaccuracy of frame-based systems like the Leksell System (Renier and Massager, 2019).

The main difference between frame-based and robotic surgery is found within the final steps of the procedure, the adjustment of the trajectory. Frame-based stereotaxy requires manual transfer of coordinates from a planning software to the frame, which might be a relevant factor for error. Some frame-based systems have the possibility to check the trajectory and the instrument used (e.g., biopsy needle) on a phantom before starting the actual surgery. This is not possible with robotic systems. In contrast, robotic surgery automatically translates planning into movement and positions the robotic arm along the planned trajectory (Spyrantis et al., 2019). Due to the automated workflow, robotic procedures might have a relevant advantage.

## Limitations

Inherent to the setup, data acquired in this experimental setting cannot be transferred to the clinical routine: the phantom does not imitate variables like the skin, skull and intracerebral structures that might cause probe deviation and therefore influence accuracy. Also, the fusion of images with different resolutions of soft tissue and bone structures, like CT and MRI, can result in deviations. There are many other procedural factors influencing the accuracy, we therefore limit the comparison to mechanical accuracy.

There are limitations in the experimental setting due to the configuration of the phantom. For acquiring the planning CT, targets are represented by a sphere with the target point being defined as the center of that sphere. For detection of accuracy, target points are represented by needle tips. Minor deviations can therefore not be excluded. Both the spheres and needle tips are metal devices, in the X-ray as well as the CT scan, metal results in artifacts, causing possible deviations on imaging. The metal rods are manually positioned in the phantom's cavities but not fixed. There is no retainer keeping them in position, the definite position of the movable rods can only be controlled manually.

Hence, the actual target point is mobile and therefore a potential source for error. Apart from the mechanical deviations, there is a possible examiner bias when determining the target point. The definition of the target point within the sphere is not automated but determined by the examiner, who is manually placing the target point into the center of the sphere depicted by the ROSA software. Also the final position of the probe's tip is manually determined and theoretically biased as the initially defined trajectory and target point are virtually depicted during this process. Future studies may utilize shape-fitting software in order to improve the process of visually determining the target point.

The imaging error is a summary of deviations occurring in the process of determining the coordinates. This includes the accuracy and resolution of the CT scan and the X-ray detecting the frame-based coordinate system for referencing as well as depicting the target. When registering the coordinate system, only minor deviations are evaluated as acceptable by the software. Both the Leksell frame and the ROSA are subject to regular calibrations, correcting fluctuations in accuracy. The permanently installed X-ray unit we used for our experiment was constructed to precisely determine the final position of a stereotactic probe during surgery. The superiority regarding image resolution of most stationary X-ray units compared to mobile units was shown before (Naji et al., 2016). With a pixel size of 0.175 mm and an image resolution index of 2 lp/mm (linepairs/mm) in both the sagittal and the anterior-posterior X-ray unit, this 3D X-ray solution is considered accurate. Nevertheless, we determined an approximation of the imaging error inherent to our experiment: We merged the planning data depicting the planned target point with the final X-ray depicting the actual position of the target point and the final position of the probe. We did not only calculate the Euclidian distance between the actual target point and the probe, but also the Euclidian distance between the planned and the actual target point depicted in the X-ray. We then calculated a mean imaging error of 0.70 mm (SD ± 0.18 mm, 95% CI 0.66–0.78 mm). This imaging error may therefore be considered a combined CT- and X-ray imaging error, including the referencing error. With an imaging error in the sub-millimeter range, we consider this X-ray unit an adequate tool for our experiment. A previous study determining the orientation angle of DBS leads confirms high accuracy of the X-ray unit (Sitz et al., 2017).

The resolution of both the imaging used for trajectory planning and of the X-ray used for analysis remained the same for both experimental procedures. While we report a high level of accuracy by employing the experimental setup described above, our findings have to be critically discussed in light of the above-mentioned imaging error. We conclude that both setups display an excellent degree of accuracy, that may however only be achieved in an experimental setup such as in our phantom study.

The results cannot be transferred to other robotic devices due to obvious differences in design and workflow.

## Conclusion

In this elaborate experimental setting, using the same imaging and referencing method, we accomplished direct comparison of frame-based and robotic accuracy. Both methods proved very precise. Assessment indicated a higher degree of accuracy in robotic procedures.

## Data Availability Statement

The raw data supporting the conclusions of this article will be made available by the authors, without undue reservation.

## Author Contributions

AS contributed to the study conception and design, was in charge of the project administration, data curation, and interpretation and wrote the original manuscript draft. TF contributed to the study conception and design, project administration, data curation and interpretation, reviewing, and editing. TW and AC contributed to the data curation, interpretation and illustration, both their doctoral theses are part of this study. HT contributed to the study conception and design, material preparation and data analysis, and commented on the manuscript. DR, AG, KL, MR, and VV-V contributed to data curation and validation. EH contributed to data interpretation and statistical analysis. FG and MC contributed to data interpretation and commented on the manuscript. All authors added intellectual content and approved of the final version of the manuscript before submission.

## Funding

AS received funding from the German Society of Neurological Surgery for this study, the Traugott-Riechert scholarship.

## Conflict of Interest

The authors declare that the research was conducted in the absence of any commercial or financial relationships that could be construed as a potential conflict of interest.

## Publisher's Note

All claims expressed in this article are solely those of the authors and do not necessarily represent those of their affiliated organizations, or those of the publisher, the editors and the reviewers. Any product that may be evaluated in this article, or claim that may be made by its manufacturer, is not guaranteed or endorsed by the publisher.
